# Visualisation of Bluetongue Virus in the Salivary Apparatus of *Culicoides* Biting Midges Highlights the Accessory Glands as a Primary Arboviral Infection Site

**DOI:** 10.1186/s12575-023-00221-2

**Published:** 2023-11-06

**Authors:** Marc Guimerà Busquets, Faye V. Brown, Simon T. Carpenter, Karin E. Darpel, Christopher J. Sanders

**Affiliations:** 1 The Pirbright Institute, Ash Road, Woking, GU24 0NF UK; 2 The School of the Biological Sciences, University of Cambridge, Mill Lane, Cambridge, CB2 1RX UK; 3 Institute of Virology and Immunology, Mittelhäusern, 3147 Switzerland; 4 Department of Infectious Diseases and Pathobiology, Vetsuisse Faculty, University of Bern, Bern, 3012 Switzerland

**Keywords:** Arbovirus, Bluetongue virus, *Culicoides*, Salivary glands, Accessory glands, Virus-vector interactions

## Abstract

**Background:**

Arthropods transmit a wide range of pathogens of importance for the global health of humans, animals, and plants. One group of these arthropod vectors, *Culicoides* biting midges (Diptera: Ceratopogonidae), is the biological vector of several human and animal pathogens, including economically important livestock viruses like bluetongue virus (BTV). Like other arthropod-borne viruses (arboviruses), *Culicoides*-borne viruses must reach and replicate in the salivary apparatus, from where they can be transmitted to susceptible hosts through the saliva during subsequent blood feeding. Despite the importance of the salivary gland apparatus for pathogen transmission to susceptible animals from the bite of infected *Culicoides*, these structures have received relatively little attention, perhaps due to the small size and fragility of these vectors.

**Results:**

In this study, we developed techniques to visualize the infection of the salivary glands and other soft tissues with BTV, in some of the smallest known arbovirus vectors, *Culicoides* biting midges, using three-dimensional immunofluorescence confocal microscopy. We showed BTV infection of specific structures of the salivary gland apparatus of female *Culicoides* vectors following oral virus uptake, related visualisation of viral infection in the salivary apparatus to high viral RNA copies in the body, and demonstrated for the first time, that the accessory glands are a primary site for BTV replication within the salivary apparatus.

**Conclusions:**

Our work has revealed a novel site of virus-vector interactions, and a novel role of the accessory glands of *Culicoides* in arbovirus amplification and transmission. Our approach would also be applicable to a wide range of arbovirus vector groups including sand flies (Diptera: Psychodidae), as well as provide a powerful tool to investigate arbovirus infection and dissemination, particularly where there are practical challenges in the visualization of small size and delicate tissues of arthropods.

## Background

Arthropod-borne viruses (arboviruses) are a taxonomically diverse group transmitted between their hosts by certain species and populations of insects and arachnids [1]. Arboviruses have emerged across the plant and animal kingdoms on multiple occasions and in some cases have evolved to form extremely complex and specific transmission cycles. One unifying feature of the group is that arboviruses are largely transmitted through the bite of an infected arthropod vector, exploiting what are usually parasitic relationships between vector and host. In the case of arboviruses of vertebrates, virus transmission involves infection and replication in both the vertebrate host and the arthropod vector. To achieve this, an arbovirus ingested in a meal of host blood, infects, and subsequently escapes the arthropod midgut, disseminates through the hemocoel and secondary organs and then infects, and escapes from the salivary glands into the saliva from which it can be transmitted to the next vertebrate host during subsequent blood-feeding [10, 16, 23]. The proportion of an arthropod species or population capable of supporting this replication and dissemination process and therefore in being at least theoretically capable of transmission is usually termed vector competence (VC) [5, 34].

Arboviruses are of considerable importance in the global health of humans, animals and plants and are prone to emergence and re-emergence events driven primarily by globalization and other forms of environmental change [4, 7, 42]. The emergence of bluetongue virus (BTV) in Europe is among the most extreme examples of this phenomenon, involving a major shift in the epidemiology of the arbovirus and establishment of endemicity in multiple countries that had never previously recorded outbreaks [20, 32, 37]. Bluetongue virus (family: *Sedoreoviridae*) is transmitted primarily by *Culicoides* biting midges (Diptera: Ceratopogonidae) and infects domestic ruminants, certain species of deer and wildlife [8, 19]. The disease caused by BTV, bluetongue (BT), is of significant economic importance in livestock husbandry in both intensive agricultural settings and subsistence farming worldwide, both through direct clinical cases and global movement restrictions imposed to limit spread [6, 12, 25, 35, 39].

The process of infection and dissemination of arboviruses in *Culicoides* has been characterized in the laboratory using a variety of direct and indirect techniques and is superficially similar to that of infection in mosquitoes (Diptera: Culicidae) [24]. This is despite these two families of insect vectors diverging over 100 million years ago and little recorded overlap in the arboviruses they are known to transmit. *Culicoides* possess several described barriers to arbovirus infection, including a mesenteron (gut) infection barrier (MIB), a mesenteron escape barrier (MEB) and a hemocoel dissemination barrier (DB) [11, 22, 26, 27]. These barriers and the ability of the virus to overcome them, lead to different scenarios of viral infection following imbibing of a blood meal from a viremic host. A proportion of individuals will clear the virus (no establishment of persistent infection), a proportion will possess an established infection but restricted to gut cells, a further number will develop an infection in the haemocoel but not fully disseminate to secondary organs, and only some individuals will develop a fully disseminated infection including the salivary glands. In contrast to mosquito studies, there is currently no evidence of the presence of barriers to virus infection and escape from the salivary glands in *Culicoides* inferred from multiple studies that have used intrathoracic infection to bypass barriers to arbovirus dissemination (e.g. [11, 30]). In mosquitoes such barriers in the salivary glands have been identified across a wide range of arbovirus-vector interactions and can play a significant role in determining the rate of virus transmission [13, 18]. It is not clear to date whether this represents a functional difference between the two families of insects in salivary gland structure and permeance to virus infection.

*Culicoides* possess two relatively large salivary gland lobes, with four to six smaller accessory glands arranged around the anterior end of each primary gland, connected via the salivary gland duct [21, 31]. In *Culicoides*, the two salivary glands typically lie within the anterior thorax, although it is not unusual for one or both glands to extend into the abdomen or head capsule [21]. Each of the two salivary gland lobes is a tube or bag-like structure of a lumen surrounded by a thin monolayer of cells [21]. The eight to twelve accessory glands of *Culicoides* are unique amongst hematophagous insects studied to date; analogous structures are not reported in the 3-lobed salivary glands of mosquitoes or single lobe glands of sand flies and black flies [17, 28, 40]. The role of these accessory glands in *Culicoides* is undetermined, although it is suggested they are involved in the production, secretion and storage of saliva components including proteins [21].

Previous studies have shown viral infection of cells of the main salivary gland lobes of *Culicoides* in cross sections of dissected salivary glands and whole insects, where dissemination to the salivary glands after intrathoracic inoculation or oral infection from feeding on a blood:virus mix has been demonstrated [3, 9, 11, 26, 27]. Detailed study of cellular and structural associations with virus infection and replication within the salivary apparatus has, however, been limited by the thin membrane structure and absence of cells in the lumen of the glands [21]. This, together with the multi-gland composition of the *Culicoides* salivary apparatus and the small size of the accessory glands, makes identification of the glands and interpretation of location and cell infection within two-dimensional cross sections of a whole insect, difficult [26, 27]. Here, we present a method to visualize the structure of these organs in 3 dimensions using immunofluorescence confocal microscopy. Using bluetongue virus as a model for *Culicoides*-borne viruses, we studied arboviral infection in the whole salivary apparatus of female *Culicoides*. Bluetongue virus infection and replication were visualized in the excised salivary apparatus of BTV-infected *Culicoides sonorensis* Wirth & Jones and *C. nubeculosus* (Meigen 1830), uncovering a potential and previously unknown key role of the accessory salivary glands of *Culicoides* in arbovirus amplification and its subsequent transmission. Visualization of virus in soft tissues, including the salivary apparatus and midgut, by confocal microscopy, linked to quantification of virus genome, presents a powerful tool for the investigation of the infection and dissemination characteristics that determine the ability of a vector to support arbovirus transmission.

## Results

### Development of a Protocol for 3D Imaging of the Salivary Apparatus of *Culicoides spp.* Using Confocal Microscopy

**Fig. 1:**
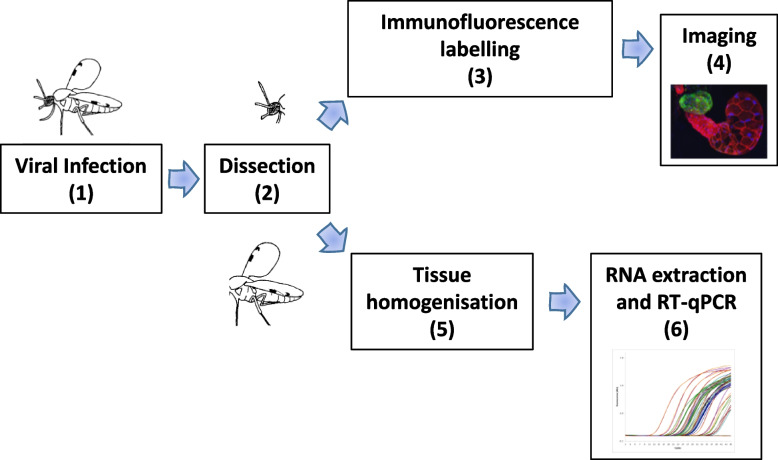
Protocol schematic to study arboviral infection in the salivary apparatus of *Culicoides spp*. **1.** Female *C. sonorensis* Wirth & Jones 1957 (PIRB -s-3 strain), or *C.* *nubeculosus* Meigen 1830 (PIRB strain) were infected by either membrane feeding on horse blood containing 7.4 log_10_ TCID_50_/mL of BTV-4 MOR2009/07 (BTV-4) or by intrathoracic inoculation with ≤ 0.2 µL at 6.4 log_10_ TCID_50_/mL of BTV-4. Female insects were selected and incubated between 5 and 15 days according to experiment (see “Results” section). **2.** After incubation, insects were anesthetized with CO_2_, and heads with the salivary apparatus (SA) still attached were dissected (and any other soft organs of interest). **3.** Dissected SA glands were then fixed with 4% paraformaldehyde, permeabilised with 0.5% Triton X-100/PBS and labelled for cellular tubulin (mouse anti-tubulin, from Sigma-Aldrich as primary antibody (ab) and anti-mouse IgG AlexaFluor™ 405 or 568, from Invitrogen as secondary ab), viral structural proteins (guinea pig anti-BTV structural proteins Orab279, from in-house, as primary ab, and anti-guinea pig IgG AlexaFluor™ 488, as secondary ab) and/or viral non-structural protein NS2 (rabbit anti-NS2 Orab1, from in-house, as primary ab, and anti-rabbit IgG AlexaFluor™ 568, as secondary ab), and cell nuclei stained with 6-Diamidino-2-phenylindole (DAPI, from Life Technologies). Labelled and stained salivary apparatus with the head still attached were mounted on a microscope slide within a gene-frame (25 µL, 10 mm x 10 mm; Thermo Scientific™) containing Vectashield® Hardset Mounting Medium (Vector Laboratories). **4.** Samples were then imaged using a Leica SP8 CLSM confocal microscope (Leica Microsystems, Wetzlar, Germany), and analyzed and exported using the Leica Application Suite X. **5.** Matching bodies to heads and SA, were homogenized using a Tissue Lyzer (Qiagen), and **6,** viral RNA was extracted using the MagMAX™ CORE Nucleic Acid purification kit and a KingFisher Flex extraction robot (ThermoFisher Scientific). Bluetongue virus RNA was detected using a Segment-10 BTV serogroup RT-qPCR assay (adapted from [15])

**Fig. 2:**
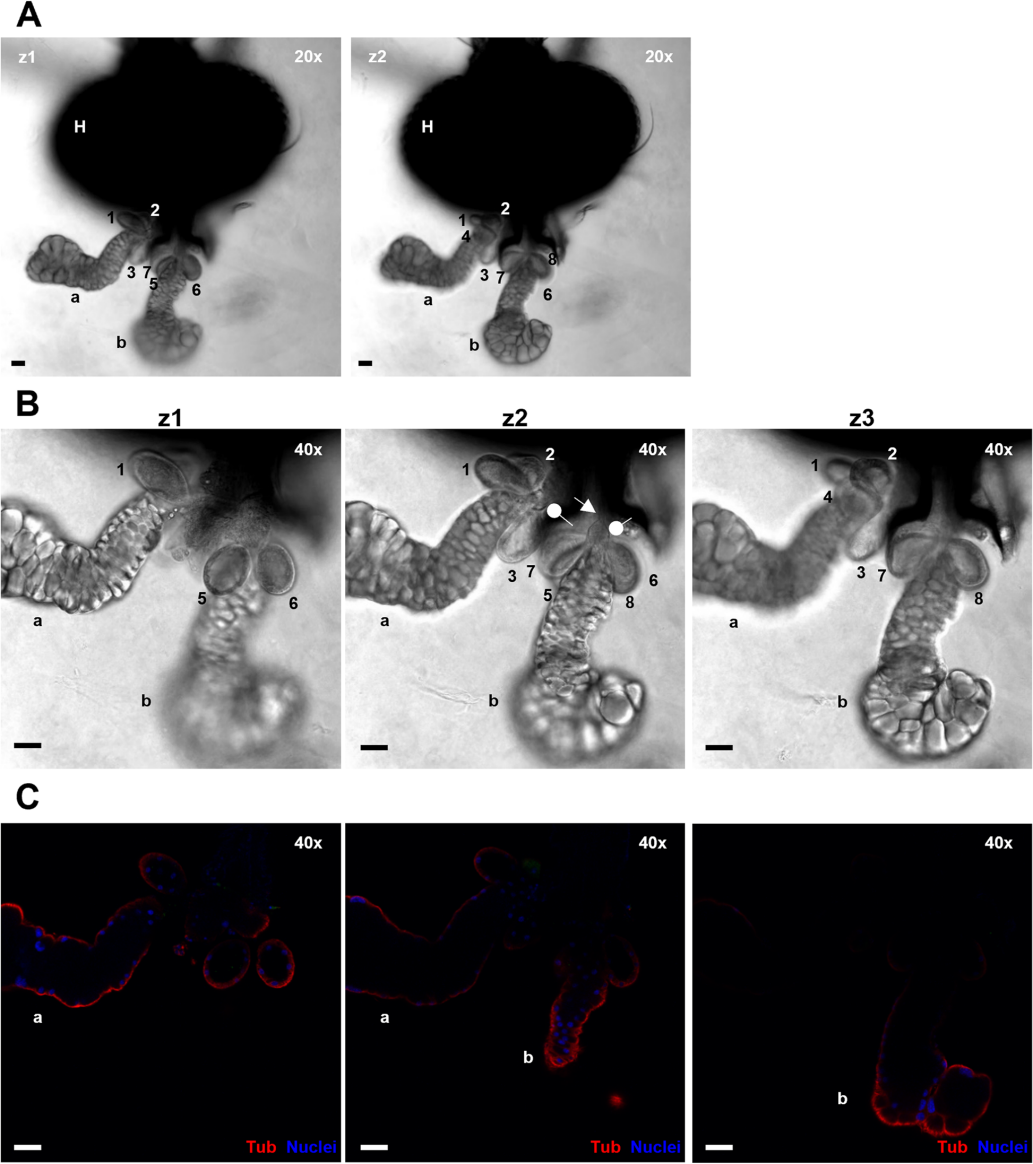
Salivary apparatus of a *Culicoides sonorensis* female. **A.** 20X magnification pictures of two planes (z1 and z2) of a head (H) with a complete salivary apparatus of a female of *C. sonorensis*, using differential interference contrast (DIC) microscopy. **B** and **C.** 40X magnification of the same salivary apparatus as in panel (**A**). Three different planes (z1 to z3) are shown using both DIC (**B**) and fluorescence confocal microscopy (**C**). In **B**, oval arrows highlight the dilation or ampulla where the salivary and accessory glands merge, and the salivary duct starts (arrow). In **C**, cell nuclei are shown in blue (DAPI staining) while tubulin is visualised in red (labelling with mouse anti-tubulin, and anti-mouse IgG AlexaFluor™ 568). In both **A** and **B** panels, two large salivary glands (**a** and **b**) and eight accessory glands (1 to 8) are distinguished. The accessory glands are sac-like shaped and arranged in groups of four as a rosette around each main salivary gland. In all panels, microscope magnification is shown on the top right corner and the scale bar represents 20 µm

A methodology was developed to visualise 3D structures/organs of small insects to investigate organ anatomy as well as visualise viral infection, with a focus on the salivary gland apparatus of *Culicoides* (Fig. 1). Two hundred and twenty-six female *Culicoides* of two species were dissected and further processed for imaging (Table 1). The complete salivary gland apparatus is composed of two salivary glands (sg) and 8 small, sac-like accessory glands (ag) and could be best processed and visualised by leaving it attached to the head during dissection (Fig. 2A).

**Table 1:** Total salivary apparatus dissections: gland recovery efficiency

	**No. of large salivary glands (sg) recovered / insect**			**No. of accessory glands (ag) recovered / insect (Across both sg)**			**Total**
	**2 sgs**	**1 sg**	**0 sgs**	**4 to 8 ags**	**1 to 3 ags**	**0 ags**	
	72 (51.8%)	54 (38.8%)	13 (9.4%)	42^a^ (30.2%)	50 (36%)	47 (33.8%)	
***C. sonorensis*** No. of insects (%)	**No. of salivary apparatus with sg but not ag**			**No. of salivary apparatus with ag but not sg**			139
	34 (24.5%)			0			
	35 (40.2%)	38 (43.7%)	14 (16.1%)	23^b^ (26.4%)	38 (43.7%)	26 (29.9%)	
***C. nubeculosus*** No. of insects (%)	**No. of salivary apparatus with sg but not ag**			**No. of salivary apparatus with ag but not sg**			87
	15 (17.2%)			3 (3.4%)			

Total number of dissected *Culicoides* (including infected and mock-infected individuals) whose salivary apparatus and bodies were processed in this study for imaging and qRT-PCR, respectively. In total, 139 *C. sonorensis* and 87 *C. nubeculosus* were processed. The number (and percentage) of salivary apparatuses from *C. sonorensis* or *C. nubeculosus* are shown in relation to the number of large salivary glands (sg) or accessory glands (ag) remaining after dissection and immunofluorescence labelling. Salivary apparatus integrity was assessed by differential interference contrast (DIC) and fluorescence confocal microscopy

^a^12 insects had at least one sg with four ags

^b^7 insects had at least one sg with four ags

**Fig. 3:**
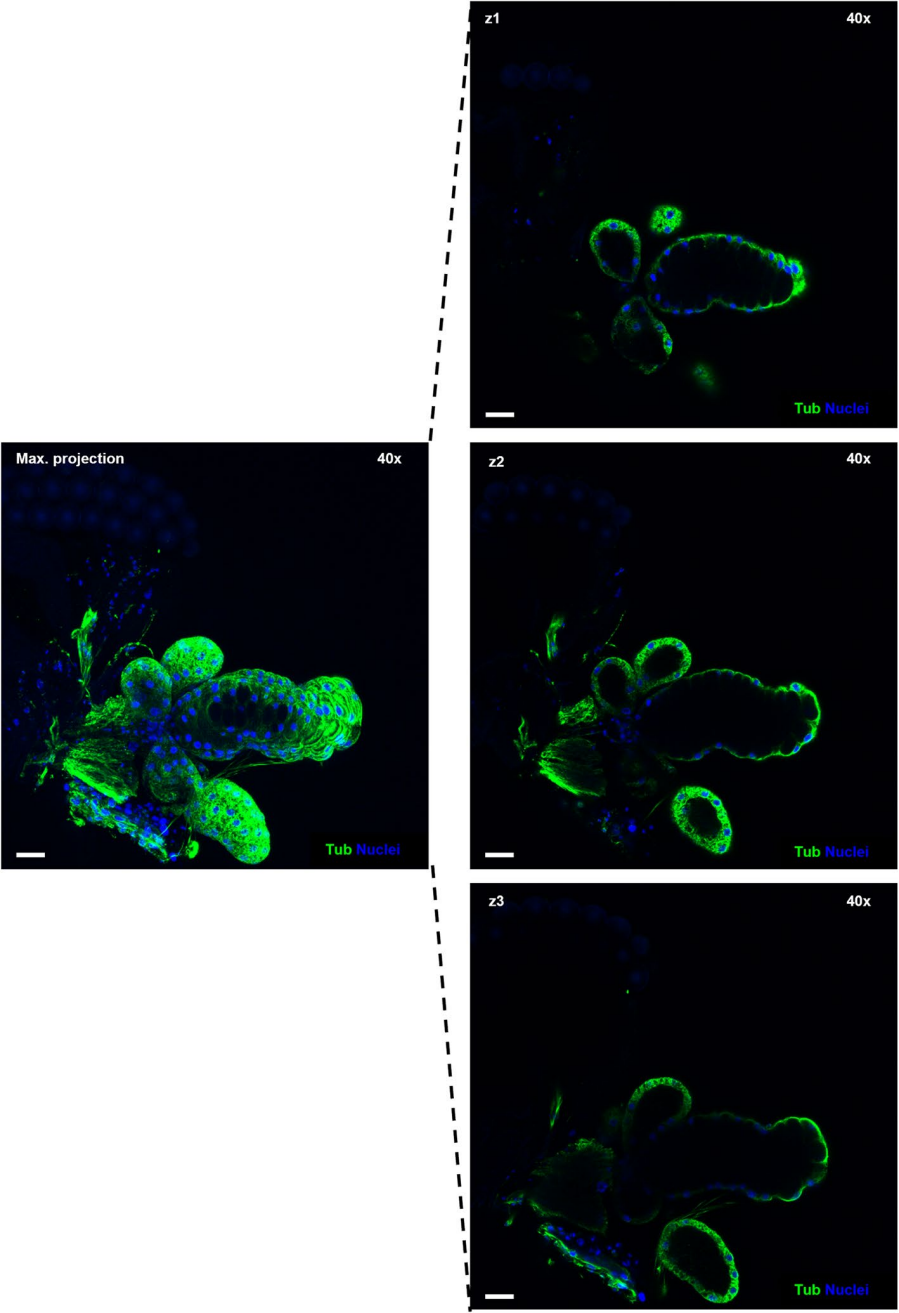
3D reconstruction of one salivary gland and associated accessory glands of a female *C. sonorensis*. Single plane images (z1-z3) show the lumen where cells are absent, surrounded by a single cell layer in both salivary gland and accessory glands. Maximum projection highlights the overall 3D structure. In all panels cell nuclei are shown in blue (DAPI staining) while tubulin is visualised in green (labelling with mouse anti-tubulin, and anti-mouse IgG AlexaFluor™ 488). In all panels, microscope magnification is shown on the top right corner and the scale bar represents 20 µm

**Fig. 4:**
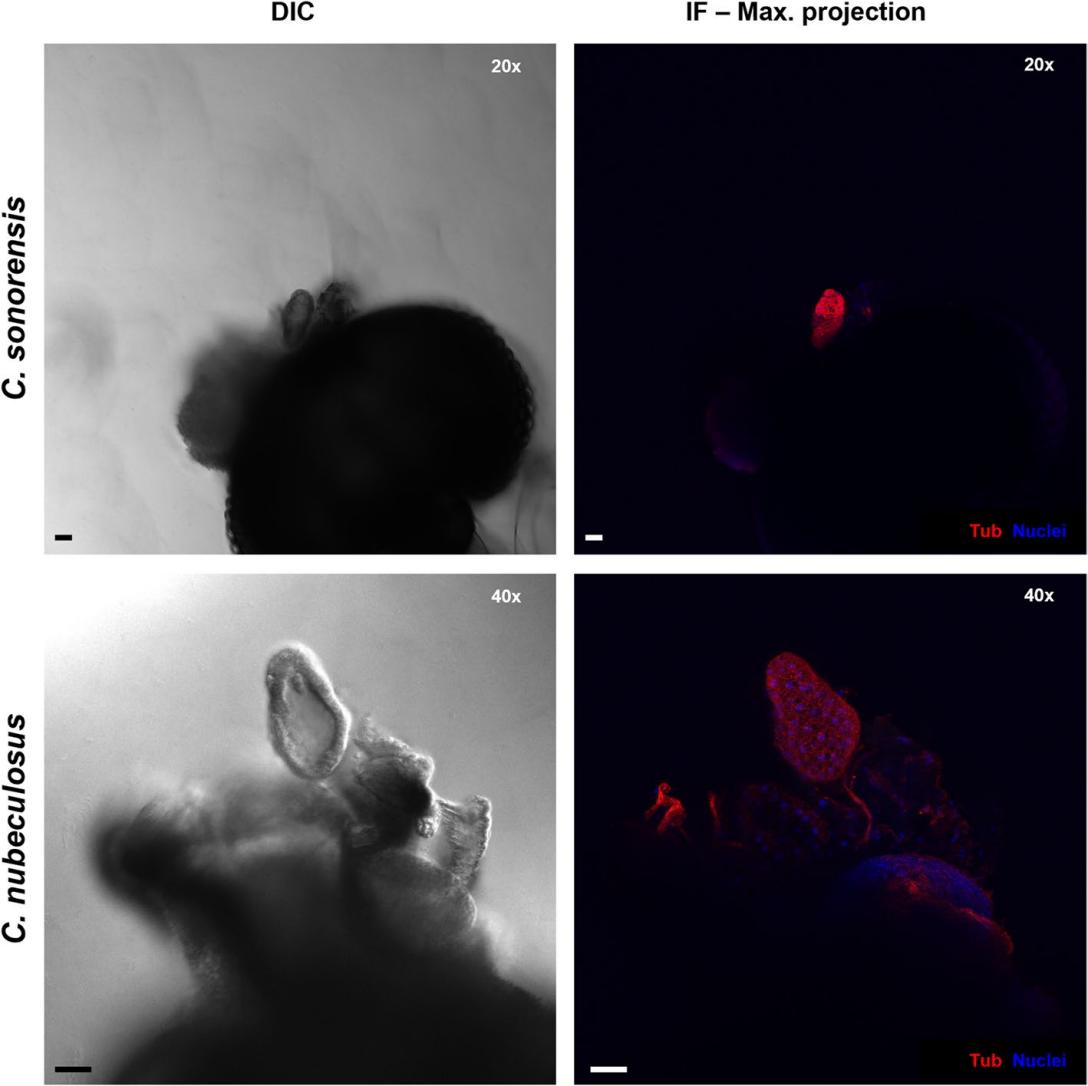
Salivary glands of males of *Culicoides sonorensis* and *Culicoides nubeculosus*. 20X and 40X magnification pictures of the salivary glands of males of *C. sonorensis* and *C. nubeculosus*, respectively. The left panels depict gland structures in one z plane by differential interference contrast (DIC) microscopy. In the right panels, gland cellular structures are shown as maximum projection images by fluorescence confocal microscopy, where cell nuclei are shown in blue (DAPI staining) and tubulin is visualised in red (labelling with mouse anti-tubulin, and anti-mouse IgG AlexaFluor™ 568). In all panels, microscope magnification is shown on the top right corner and the scale bar represents 20 µm

Immunolabeling of cellular tubulin and staining of cell nuclei of the head-salivary apparatus was successful and showed both gland types were composed of a single cell layer surrounding a central lumen (Figs. 2 and 3). Combining differential interference contrast (DIC) and confocal fluorescence microscopy, all parts of the salivary apparatus were identifiable, i.e., salivary glands (sg), accessory glands (ag), ampulla, and salivary duct (Fig. 2B). For *C. sonorensis*, the maximum number of glands observed in females was two main glands with four accessory glands joined to each main gland (Fig. 2), confirming previous findings by Perez de Leon et al. [31] (*Culicoides sonorensis* was previously undifferentiated within the *C. variipennis* group). Three-dimensional visualisation of the salivary glands was also successfully achieved using a series of Z-stack images (Fig. 3). An identical number and arrangement of glands were observed in *C. nubeculosus*. These structures in the female insect contrasted with the reduced salivary gland lobes observed in male specimens, which also lacked accessory glands (Fig. 4).

### The Accessory Glands of *Culicoides sonorensis* Midges are a Primary Bluetongue Virus Replication Site within the Salivary Apparatus

**Fig. 5:**
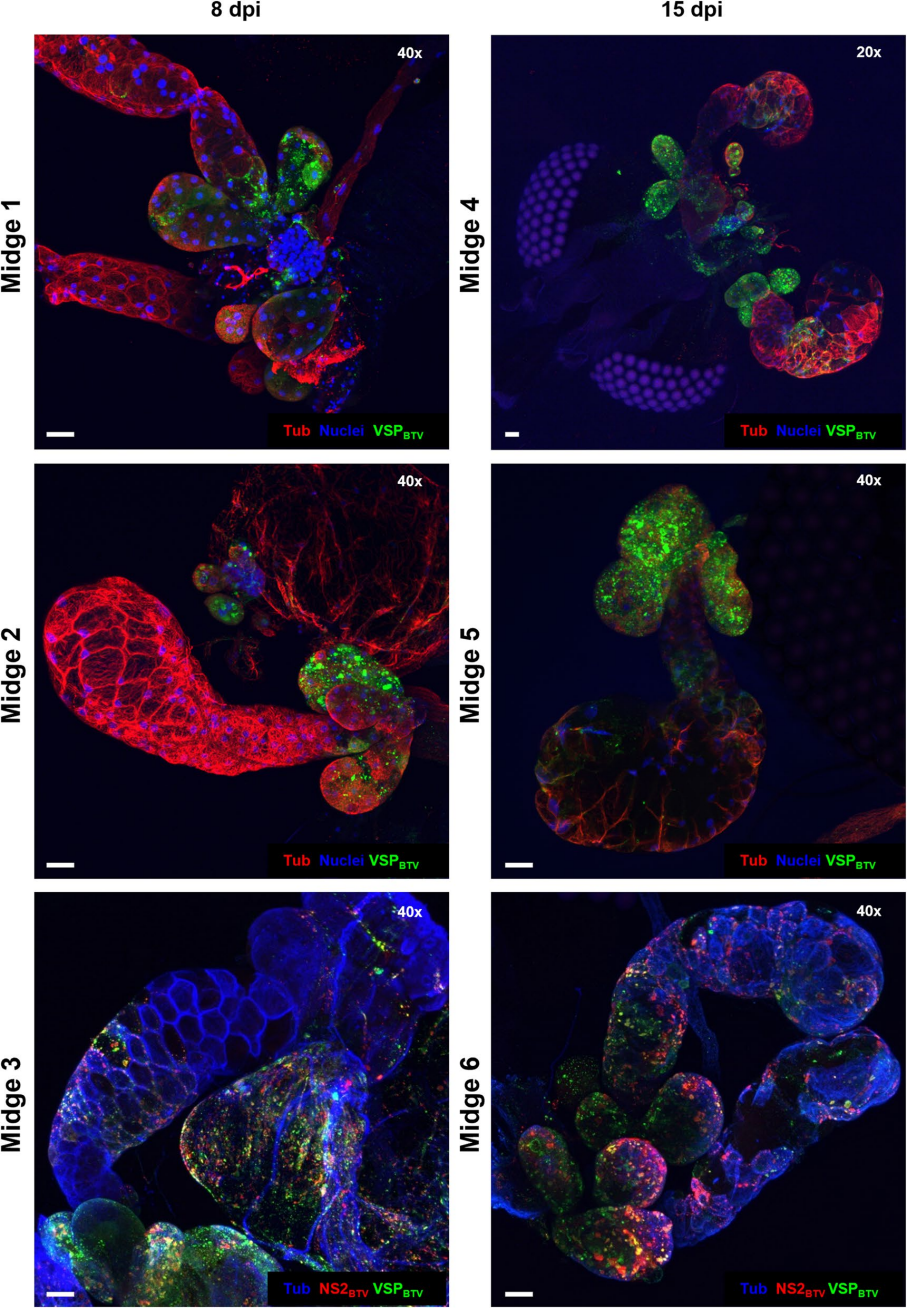
Bluetongue virus infection in salivary apparatus of females of *Culicoides sonorensis* infected by blood feeding. Six representative images of infected salivary apparatuses from six different females of *C. sonorensis*. All images are maximum projections of at least 50 stack images. Midges 1 to 3 are a representative of BTV infection after 8 days post infection (dpi), while midges 4 to 6 are a representative of infection at 15 dpi. In midges 1, 2, 4 and 5 cell nuclei are shown in blue (DAPI staining), cellular tubulin is visualised in red (labelling with mouse anti-tubulin, and anti-mouse IgG AlexaFluor™ 568) and BTV viral structural proteins (VSPs) is visualized in green (labelling with in-house antibody Orab279 and anti-guinea pig IgG AlexaFluor™ 488). Midges 3 and 6 show cellular tubulin in blue (labelling with mouse anti-tubulin, and anti-mouse IgG AlexaFluor™ 405), BTV VSPs in green (labelling with in-house guinea pig antibody Orab279 and anti-guinea pig IgG AlexaFluor™ 488), and BTV non-structural protein 2 (NS2) in red (labelling with in-house rabbit antibody Orab1 and anti-rabbit IgG AlexaFluor™ 568). In all panels, microscope magnification is shown on the top right corner and the scale bar represents 20 µm

**Fig. 6:**
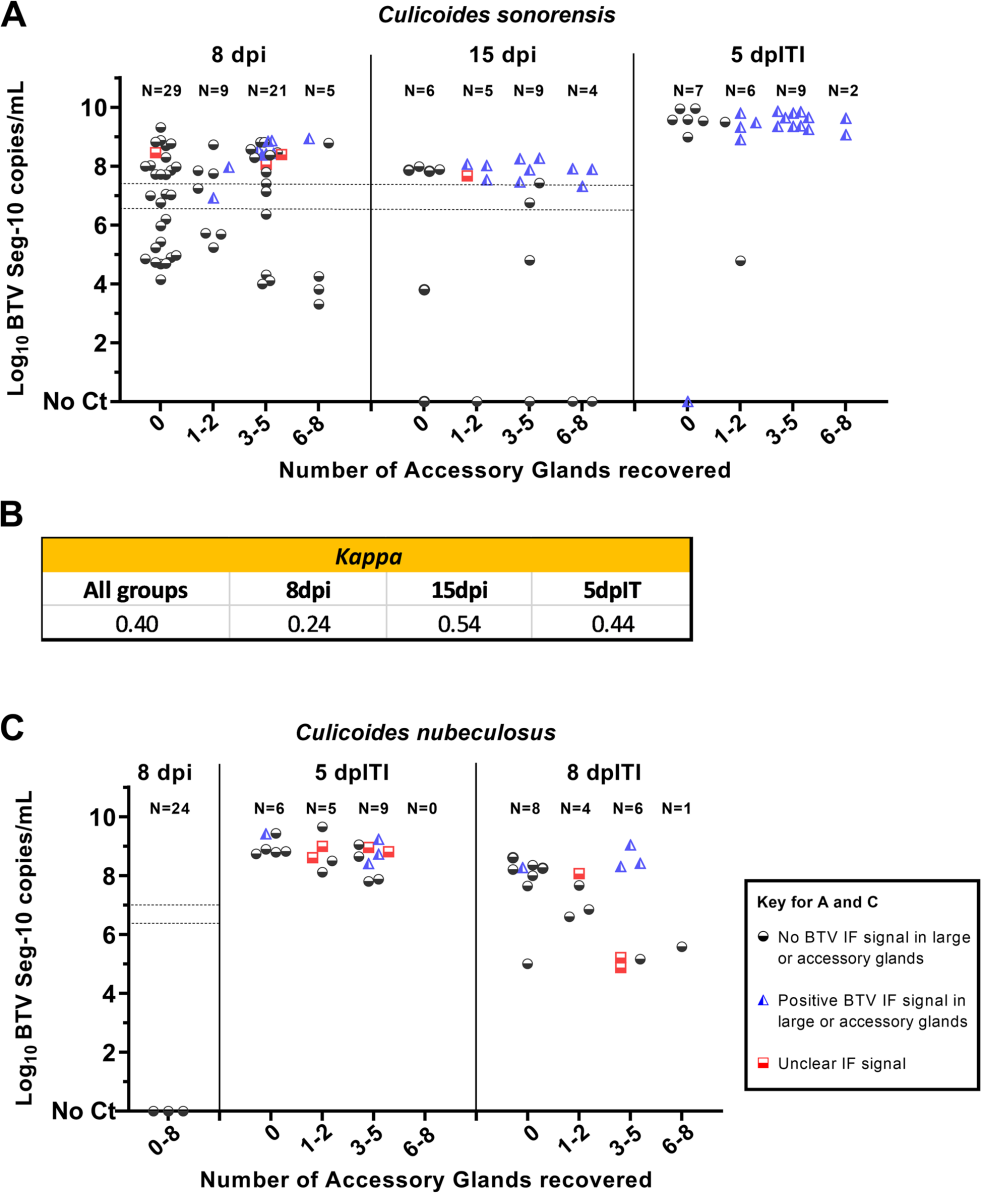
Visualisation of BTV infection in the salivary glands relates to increasing quantities of viral RNA in the body. **A**. Bluetongue virus segment 10 (Seg-10) copies / mL in *Culicoides sonorensis* at 8 or 15 days post oral infection (dpi) and 5 days post intrathoracic infection (dpITI); **B**. In *C. sonorensis* with at least 1 accessory gland (ag) recovered, agreement between BTV detection in the salivary glands by immunofluorescence (IF) labelling and BTV genome detection in the body by qRT-PCR. A *Kappa* (*K*) test was performed in all infection groups together or individually. *K* < 0.4 = poor agreement, 0.4 ≤ *K* ≤ 0.75 = moderated to good agreement and *K* > 0.75 = excellent agreement. **C**. BTV Seg-10 copy numbers / mL in *Culicoides nubeculosus* at 5 or 8 dpITI, or 8 dpi after oral feeding. In **A** and **C**, viral copy numbers were obtained after carrying out a BTV-specific qRT-PCR assay targeting segment 10 (adapted from Hofmann et al. [15]) on viral RNA extracted from homogenized individual *Culicoides* bodies, except for 8 dpi in **C**, where *C. nubeculosus* were processed in three pools of eight insects each. For each insect, BTV genome copies are plotted against the number of ag recovered after dissection and immunolabelling of the head-salivary apparatus complex. Absence of BTV IF signal in the salivary glands (including main lobes and ag) is shown by circles/black, detection of BTV IF signal in the salivary glands is shown by triangles/blue and unclear IF signal is shown by squares/red. Finally, dotted lines in **A** and **B** comprise the range of viral genome copies obtained from *Culicoides* homogenized at 0 days post oral infection

Infection of the salivary glands of competent *Culicoides* species is considered paramount for viral transmission to susceptible hosts. However, previous research on salivary apparatus infection is limited, and has focused on the main salivary glands with no mention of the accessory glands [3, 9, 11, 26, 27]. Mimicking a natural route of infection, female *C. sonorensis* were allowed to blood-feed through a membrane on BTV-spiked blood. Engorged specimens were then sorted and incubated for 8 days prior to head-salivary apparatus dissection and immunolabelling. Confocal microscopy was used successfully to visualise BTV infection of the salivary apparatus at 8 days post infection (dpi) in 26% of the *Culicoides* with at least one accessory gland recovered (Figs. 5 and 6A). Moreover, by identifying viral structural proteins (VSPs) and the non-structural protein 2 (NS2) (though labelling with respective antibodies) the methodology was able to highlight both viral presence and replication localised in the salivary apparatus (Fig. 5). Although BTV proteins were observed in the main salivary glands, the infection was typically localised to a few loci of infected cells and was not disseminated through the whole gland. In contrast, when BTV infection of the accessory glands was observed, many loci of infected cells were seen across the whole accessory gland (Fig. 5). Increasing the incubation time to 15 dpi did not lead to any apparent difference in BTV infection of the salivary apparatus (Fig. 5), remaining primarily in the accessory glands and with only reduced and localised infection in the main lobes.

**Fig. 7:**
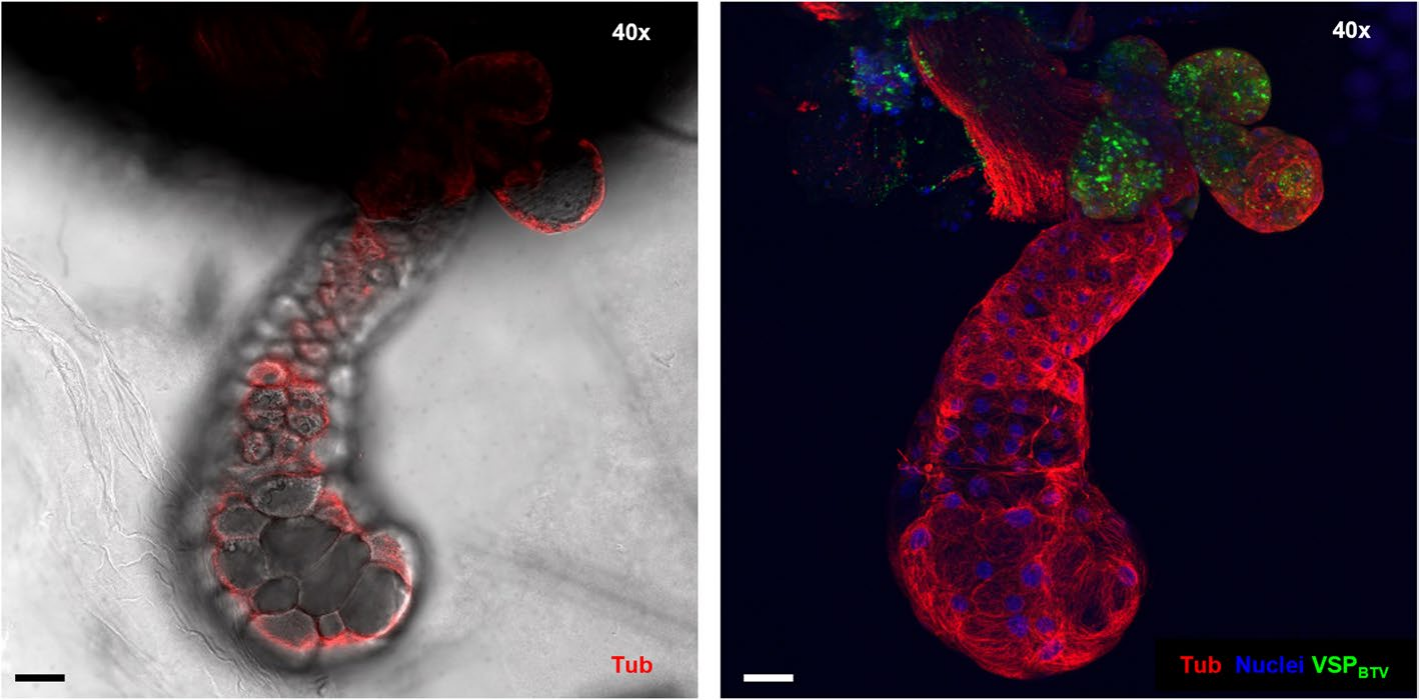
Bluetongue virus infection in salivary apparatus of a female *Culicoides sonorensis* following intrathoracic inoculation of BTV. Representative image at 5 dpITI of the salivary apparatus of a female *C. sonorensis* midge. Image on the left shows one plane taken with differential interference contrast microscopy and cellular tubulin is shown in red (labelling with mouse anti-tubulin, and anti-mouse IgG AlexaFluor™ 568). Image on the right is a maximum projection of at least 50 stack images where cell nuclei are shown in blue (DAPI staining), cellular tubulin is visualised in red (labelling with mouse anti-tubulin, and anti-mouse IgG AlexaFluor™ 568) and BTV viral structural proteins (VSPs) is visualized in green (labelling with in-house antibody Orab279 and anti-guinea pig IgG AlexaFluor™ 488). In both panels, microscope magnification is shown on the top right corner and the scale bar represents 20 µm

To infect *Culicoides species* with low vector competence or when utilising virus strains with a low ability to replicate and disseminate fully in *Culicoides*, direct intrathoracic inoculation (ITI) of *Culicoides* with virus-infected cell supernatant has been commonly used [30, 36]. ITI bypasses mesenteron infection (MIB) and escape barriers (MEB) found in the vector and typically results in 100% of surviving individuals being able to transmit the virus (100% vector competence) and hence, it is commonly assumed that ITI would result in the infection of the salivary apparatus of all individuals. Here we investigated if individual *Culicoides* females IT-inoculated with BTV demonstrated the same topology of salivary gland apparatus infection as observed for orally infected individuals. Bluetongue virus was observed in the salivary apparatus of most of individuals (88%) where at least one accessory gland was recovered (Figs. 6A and 7). The virus was observed predominantly in the accessory glands, as in the case of *C. sonorensis* that were infected using an oral route (Fig. 7). The pattern of BTV infection in the salivary apparatus did not differ amongst infection groups (8 or 15 days post oral infection, or 5 dpITI). In most individuals (independently of the infection route and incubation period), BTV-infected and non-infected glands were observed within the same salivary apparatus (Figs. 5 and 7), with few insects showing BTV infection in all the glands recovered. In some BTV-positive cases of individuals with at least one accessory gland recovered, infection was only in the accessory gland/s but not in its/their respective main salivary gland. But where main salivary glands were infected, associated accessory glands were always infected. These findings show for the first time that the accessory glands of *Culicoides* are highly permissible to arboviral infection, with evidence that they might act as a primary site for BTV replication within the salivary apparatus of vector *Culicoides spp*.

### Detection of Virus in the Salivary Apparatus by Immunolabelling is Consistent with Viral Genome Quantities in the Body

The bodies of all dissected *Culicoides* were homogenised, total RNA was extracted and BTV genome detected and quantified using a BTV-specific qRT-PCR [15]. As observed in studies of *Culicoides* vector competence for BTV [14, 33, 36], in groups infected by oral feeding, insects separate into three defined populations: those with no viral RNA (vRNA) detected (no Ct); individuals with lower amounts of vRNA (≤ 4.00E + 07 genome copies/body, equivalent to a Ct value of ≈25 in our assay) and deemed to not support virus transmission (hence not vector competent), and those with greater quantities of vRNA (≥ 4.00E + 07 genome copies/insect) and deemed vector competent (Fig. 6A). Linking BTV RNA quantification in the body of insects with at least one accessory gland (ag) recovered to confocal microscopy observations in the salivary apparatus of each individual, showed that in all three groups of *C. sonorensis* studied (8 and 15 days post oral infection, and 5 dpITI) all specimens with an infected salivary apparatus, except one, had higher viral copies in the body (Fig. 6A). However, not all *C. sonorensis* with high quantities of vRNA in the body had an observable BTV infection in the salivary apparatus under confocal microscopy. This was particularly observed in the group assessed at 8 days post oral infection. Combining all groups, the percentage of individuals with high quantities of vRNA in their bodies was 66% when calculated by qRT-PCR. This contrasts with 49% of insects with BTV detected in the salivary apparatus by immunofluorescence labelling. A *Kappa* test in individuals with at least one ag recovered (Fig. 6B) showed an overall fair agreement between both techniques (*Kappa* = 0.40), with a moderate agreement in groups assessed at 15 days post oral infection (*Kappa* = 0.54) and 5 dpIT infection (*Kappa* = 0.44).

### Bluetongue Virus Infection in Non-vector *Culicoides* Species

**Fig. 8:**
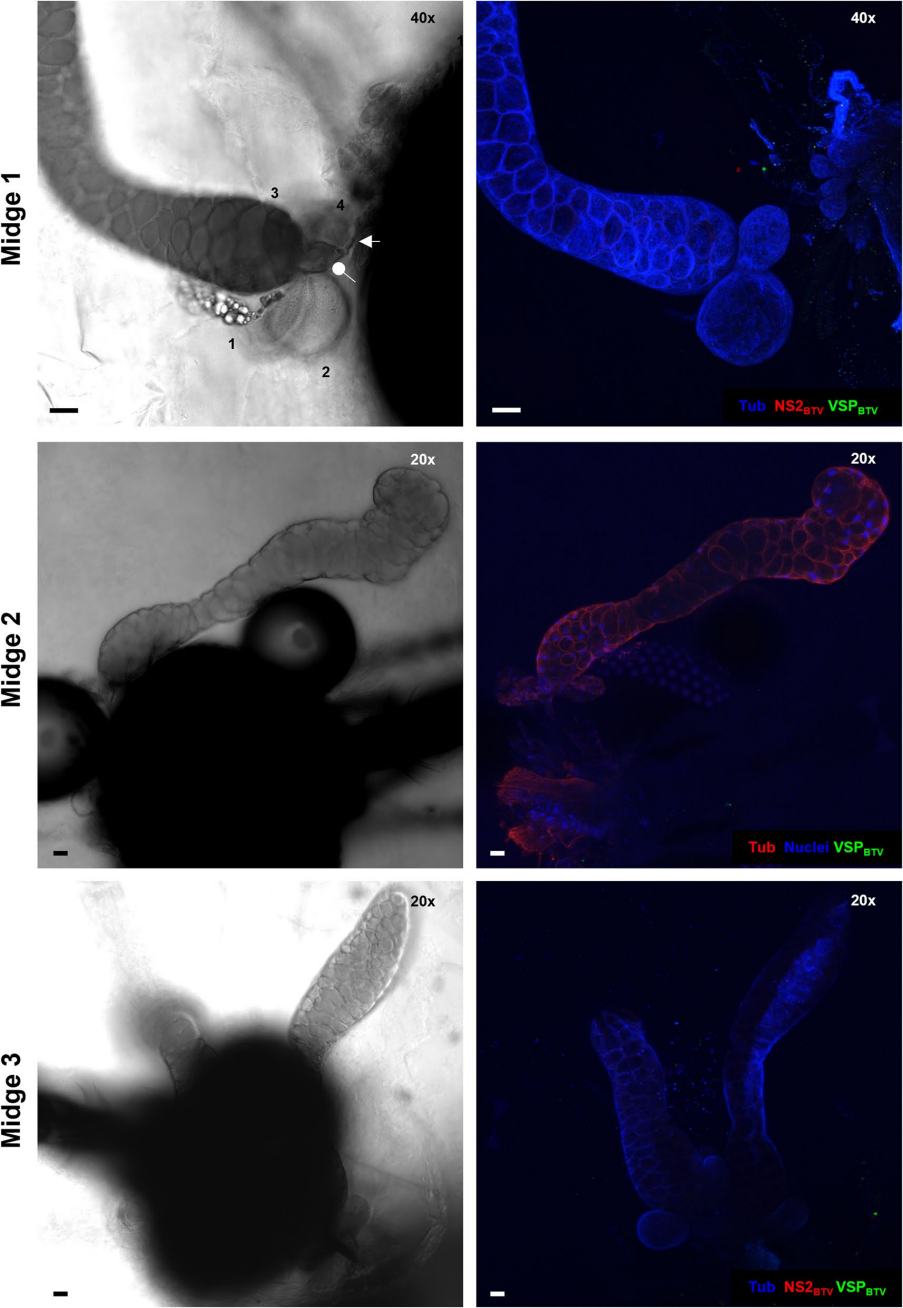
Absence of BTV infection in salivary apparatus of *Culicoides nubeculosus* fed with an infectious bloodmeal. Three representative images of salivary apparatus from three different females of *C. nubeculosus* at 8 days post infection. Images on the left show one plane taken with differential interference contrast (DIC) microscopy. Images on the right are maximum projection of at least 50 stack images. In midge 1 and 3, cellular tubulin is shown in blue (labelling with mouse anti-tubulin, and anti-mouse IgG AlexaFluor™ 405), BTV viral structural proteins (VSPs) in green (labelling with in-house guinea pig antibody Orab279 and anti-guinea pig IgG AlexaFluor™ 488), and BTV non-structural protein 2 (NS2) in red (labelling with in-house rabbit antibody Orab1 and anti-rabbit IgG AlexaFluor™ 568). In midge 2, cell nuclei are shown in blue (DAPI staining), cellular tubulin is visualised in red (labelling with mouse anti-tubulin, and anti-mouse IgG AlexaFluor™ 568) and BTV VSPs are visualized in green (labelling with in-house antibody Orab279 and anti-guinea pig IgG AlexaFluor™ 488). BTV proteins were not observed in any of the midges. In midge 1, four accessory glands around one main salivary gland are numbered and the ampulla (oval) and salivary duct (arrow) can be observed. In all panels, microscope magnification is shown on the top right corner and the scale bar represents 20 µm

Infection in the salivary apparatus of female *Culicoides nubeculosus*, a species that is largely refractory to BTV infection [41], was investigated. Bluetongue virus was not detected in the salivary apparatus of any of the twenty-four individuals fed with a blood:virus mix (Fig. 8). Analysis by qRT-PCR demonstrated viral RNA was not present in the bodies (Fig. 6C), showing BTV had been unable to establish an infection of the midgut. Infecting *C. nubeculosus* with BTV using ITI demonstrated high quantities of vRNA in all individuals by 5 dpITI, demonstrating that BTV was able to replicate if the midgut barriers were overcome. Despite this, BTV was found in the salivary apparatus of only 21% (3 out of 14) of *Culicoides* with at least one accessory gland recovered (Fig. 6C). Increasing the incubation period to 8 days altered the distribution of BTV RNA positive individuals, with a small group of individuals with lower quantities of vRNA observed (Fig. 6C) (in addition to a larger group with high quantities of vRNA). Whether this is due to an inefficient IT infection, or that with longer time periods anti-viral mechanisms of *C. nubeculosus* are able to reduce viral replication, needs to be further investigated. Nevertheless, the overall IF detection of BTV in the salivary glands at 8 dpITI did not change substantially compared to the 5 dpITI group (27% at 8dpITI vs 21% at 5dpITI), although 60% of the *C. nubeculosus* with high vRNA in the body had observable virus in the salivary apparatus (Fig. 6C).

### Investigating BTV Infection in Additional *Culicoides* Tissues

**Fig. 9:**
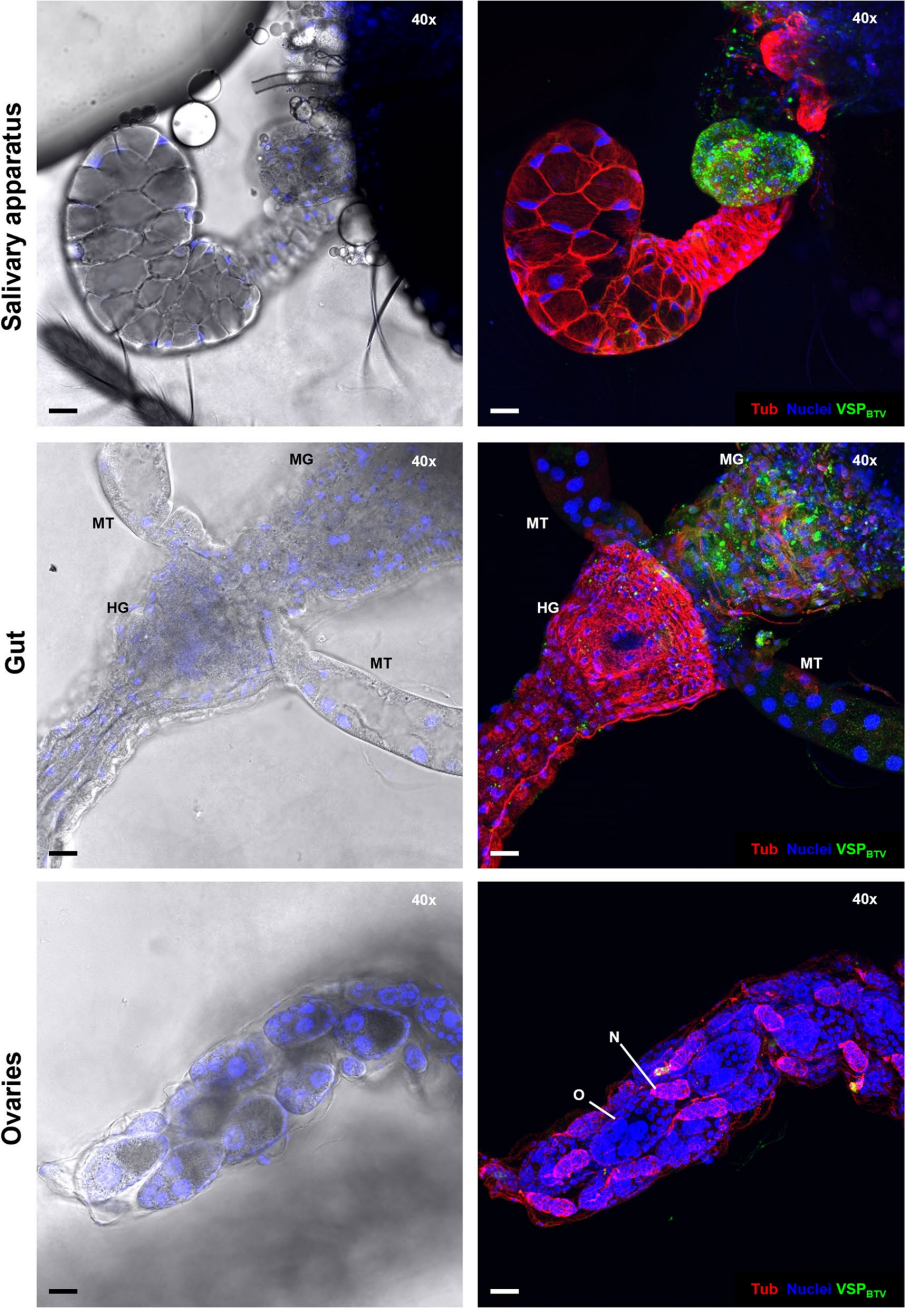
Bluetongue virus infection can be studied in several organs of the same *Culicoides sonorensis*. Salivary apparatus, gut and ovaries of the same *C. sonorensis* female at 8 days post oral infection with BTV. Images on the left show one plane taken with differential interference contrast (DIC) microscopy and show cell nuclei in blue (DAPI staining). Images on the right are immunofluorescence (IF) maximum projections of at least 50 stacked plane images, where cell nuclei are shown in blue (DAPI staining), cellular tubulin is visualised in red (labelling with mouse anti-tubulin, and anti-mouse IgG AlexaFluor™ 568) and BTV structural proteins (VSPs) are visualized in green (labelling with in-house antibody Orab279 and anti-guinea pig IgG AlexaFluor™ 488). In the salivary apparatus, BTV infection can be observed in the accessory glands, but not in the main salivary gland. In the gut, MT show the Malpighian tubules, HG show the hind gut, and MG show the midgut. The latter is infected by BTV. In the ovaries, ovarioles can be observed, which contain egg cells or oocytes (O) and nurse cells (N). BTV was detected outside the ovarioles, but not within. In all panels microscope magnification is shown on the top right corner and the scale bar represents 20 µm

Our methodology further allowed us to investigate BTV infection in different tissues of the same *Culicoides* insect (Fig. 9). Soft tissues including the gut and ovaries of a *C. sonorensis* fed on BTV-spiked blood were dissected together with its respective salivary apparatus. In the example shown (Fig. 9), following immunolabeling as described, BTV was detected in the salivary glands at 8 dpi, demonstrating complete BTV dissemination. As described throughout this study, BTV was found in the accessory glands, with no virus detected in the main salivary gland in this case. When analyzing the gut, BTV was detected in the midgut, but not in the hindgut or Malpighian tubules. Finally, BTV was detected in the ovarian sheath, but not within the ovarioles where oocytes and nurse cells are found. This agrees with previous laboratory studies inferring that vertical transmission of BTV does not occur in *C. sonorensis* [29].

## Discussion

In this study we have developed a novel protocol that has enabled three-dimensional visualisation of arbovirus infection in the salivary glands of vectors of approximately three-millimetre total body length. This technique would be applicable to a wide range of arbovirus vector groups including sand flies. We used the technique to explore arbovirus infection of the salivary gland apparatus of female *C. sonorensis* with BTV. This approach allowed us to visualise for the first time BTV infection of specific structures of the salivary gland apparatus of female *Culicoides* vectors following oral virus uptake, as well as discover strong evidence that, although all the glands constituting the salivary apparatus can harbour virus, the accessory glands are a primary and hitherto unsuspected site for BTV replication.

### Three-dimensional Immunolabelling and Imaging of Whole Dissected Salivary Apparatus and Heads Facilitates Identification of all the Glands and Location of Viral Infection

**Fig. 10:**
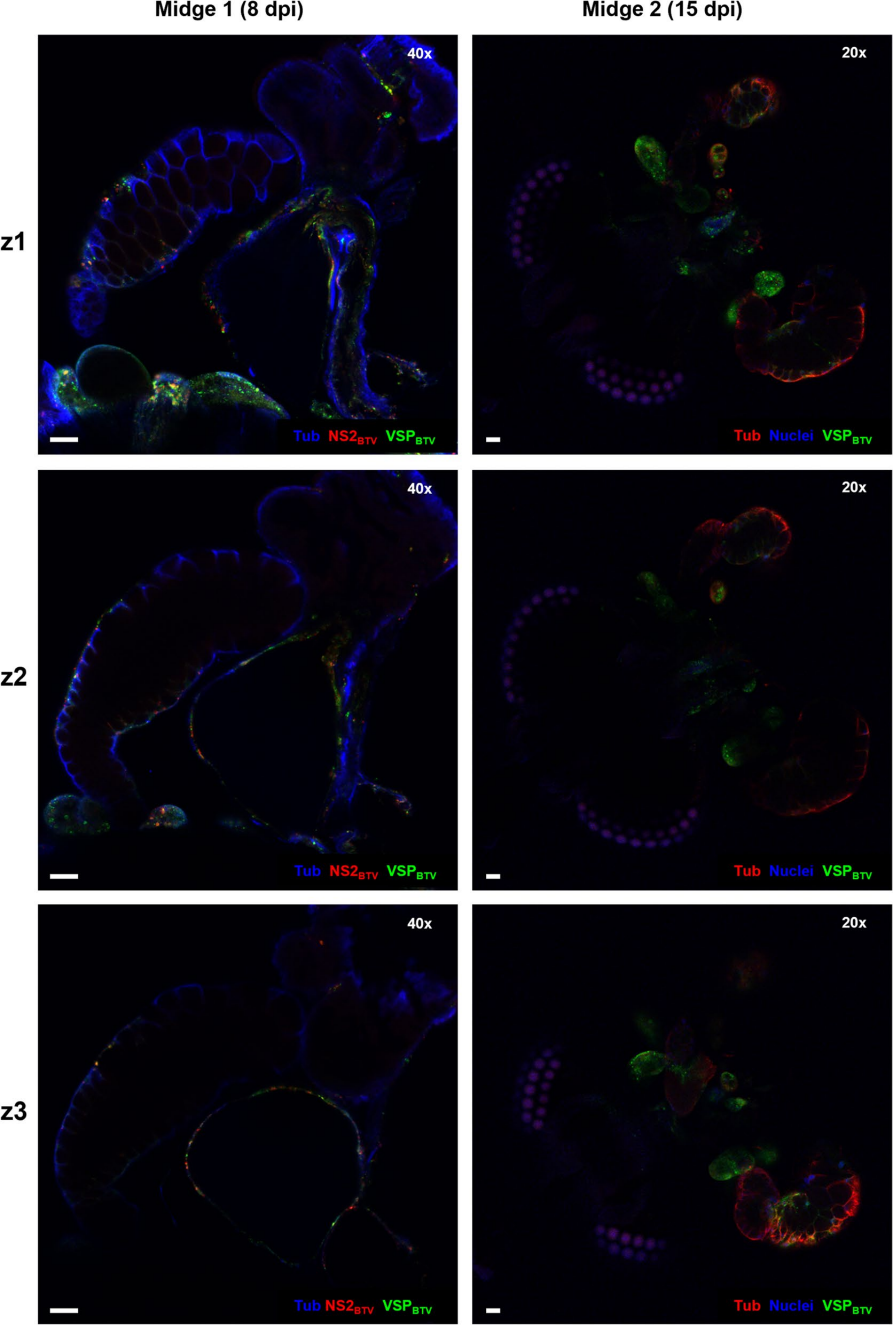
Detecting BTV infection in single planes of salivary apparatus of female *Culicoides sonorensis*. Example of single planes (z1 to z3) for two of the insects shown in Fig. 5. Viral presence and replication in the main lobe can be missed when analyzing single planes/sections due to the high ratio of lumen:cellular structures. Equally, accessory glands are hard to identify. In midge 1, cellular tubulin is shown in blue (labelling with mouse anti-tubulin, and anti-mouse IgG AlexaFluor™ 405), BTV viral structural proteins (VSPs) in green (labelling with in-house guinea pig antibody Orab279 and anti-guinea pig IgG AlexaFluor™ 488), and BTV non-structural protein 2 (NS2) in red (labelling with in-house rabbit antibody Orab1 and anti-rabbit IgG AlexaFluor™ 568). In midge 2, cell nuclei are shown in blue (DAPI staining), cellular tubulin is visualised in red (labelling with mouse anti-tubulin, and anti-mouse IgG AlexaFluor™ 568) and BTV VSPs are visualized in green (labelling with in-house antibody Orab279 and anti-guinea pig IgG AlexaFluor™ 488). In all panels, microscope magnification is shown on the top right corner and the scale bar represents 20 µm

In contrast to mosquitoes, the anatomy and histology of the salivary apparatus of *Culicoides* has not received substantial attention, with only two studies describing it in detail [21, 31]. Consequently, viral infection of the salivary apparatus of *Culicoides* has also been poorly studied, with few studies undertaken to date [3, 9, 11, 26, 27]. These studies were reliant on sectioning dissected salivary glands or whole insects prior to staining and imaging. The small size of *Culicoides* midges, the multi-gland composition, single cell layer epithelia ([21, 31], and this study) and fragility of salivary and accessory glands (Table 1) [21], makes microscopic identification of gland structure and infection in a single plane extremely challenging (Fig. 10). This is reflected by previous studies focusing on infection in the main salivary glands and failing to identify or mention the accessory glands and their potential role in virus transmission.

Our visualisation technique, although requiring skilled dissection, facilitates processing of a greater number of samples than cryo-sectioning, and presents the whole tissue or organ in a manner that reveals detailed structure as well as virus infection. It therefore has the potential to demonstrate the location of key interactions between insect tissues and arboviruses, including specific infection loci and therefore allude to mechanisms of barriers to infection. This would complement cryo-sectioning approaches required for the study of harder, chitin-rich structures (such as the head capsule) and hard to dissect structures (such as the fat body).

### Accessory Glands as a Primary Site for BTV Replication within the Salivary Apparatus of Female *Culicoides* Midges and their Potential Role for Onward Virus Transmission

Previous studies that examined viral infection of salivary glands of *Culicoides* mostly focused on intrathoracic inoculation of virus to by-pass midgut infection barriers present in *Culicoides* and ensure infection of the salivary glands [3, 9, 11]. Here we were able to visualise BTV infection of specific structures and locations in the salivary apparatus of female *Culicoides* midges following an oral route of infection and show that high quantities of viral RNA in the body are correlated to viral presence in the salivary apparatus. In infected accessory glands, BTV was detected throughout the gland cell-layer, whereas only in localised loci (if at all) of the main salivary glands. This strongly suggests that the accessory glands are highly permissible to arboviral infection and are a primary site of BTV replication within the salivary apparatus of female *Culicoides*, regardless of the route of infection. Crucially for onwards transmission on biting, the accessory glands of *Culicoides* are linked via the salivary duct to the main lobe. Accessory glands are also only found in female *Culicoides* and could potentially serve as temporary reservoirs for the secretion components of the larger salivary glands as well as actively secrete saliva components that differ in nature from those produced in the main salivary glands [21]. These associations allow us to hypothesise a potentially fundamental role of the accessory glands for transmission of BTV. Further work is required to determine if infection of the accessory glands is the causal factor in the lack of salivary gland barriers to infection in *Culicoides* and to determine if accessory gland infection is indicative of the ability of an individual to transmit a virus to a ruminant host.

### Vector Competence Studies

The dynamics of viral infection in arthropod-vectors are complex and generally poorly understood. Within a same vector *species* (*spp.*) and same virus *spp.*, several outcomes or scenarios of infection can arise as a result of the interplay of vector, virus and environmental factors. For example, vector genetic factors cause insect-to-insect variability to infection susceptibility; viral genetic factors bring differences amongst different strains of the same viral *spp.*, and environmental factors can influence both vector susceptibility to infection and virus ability to infect. One of the vector phenotypes with major consequences for disease transmission is vector competence [5, 26, 27]. Studies on vector competence can be costly and hard to perform since they require demonstration of transmission, or proof of viral presence in the insect saliva. The agreement observed between high quantities of viral RNA in the body and detection of viral proteins in the salivary glands validate, at least partially, previous studies where vector competence ratios were inferred by associating higher viral copies in the midge (by qPCR) with transmission or virus in the saliva [5, 26, 27, 41].

### Applications and Ideas

This technique also facilitates investigation of arbovirus infection and dissemination in different insect tissues including the midgut. Through using a similar process of virus quantification and IF microscopy, hypothesised midgut infection and escape barriers may be explored and visualised. The technique will also facilitate further comparative studies of infection dynamics between virus strains of the same species, and other *Culicoides*-borne viruses (e.g., Schmallenberg virus, African horse sickness virus and Oropouche virus which infects and causes disease in humans). One potential limitation is the as yet undefined sensitivity or limits of virus detection, as one would expect an assay reliant on visualisation of bound antibody is less sensitive than detection using molecular amplification techniques.

## Conclusions

Our work has revealed the accessory glands of *Culicoides*, an arthropod vector of important animal and human pathogens, as a novel site of virus-vector interaction with relevance to onward pathogen transmission to mammalian hosts. Our approach will allow further characterisation of this previously unknown role of the accessory glands. In addition, the study of infection dynamics in other soft tissues like the midgut can be examined using the technique. These approaches will be critical to enhance our understanding of *Culicoides*-virus interactions and underlying mechanisms of vector competence as well as *Culicoides*-virus-mammalian host interactions including the impact of *Culicoides* saliva on mammalian host infection. Our research will, therefore, be of specific interest to the BTV and related *Orbivirus* research community, but also to the wider fields of vector-borne diseases and arbovirus-vector interactions.

## Methods

### Virus

Bluetongue virus (BTV) isolate BTV-4 MOR2009/07 (cell passage KC1), was obtained from the *Orbivirus* Reference Collection (ORC) at The Pirbright Institute, UK (https://www.reoviridae.org/dsRNA_virus_proteins/ReoID/btv-4.htm#MOR2009/07, accessed on 13^th^ June 2023) and was previously shown to infect *Culicoides sonorensis* at high rates [36]. Working stocks of the virus were generated by two additional propagations on *Culicoides*-derived KC cells as previously described [38] and kept at + 4 °C. Infectivity of the viral working stocks was determined by fluorescent Tissue Culture Infective Dose 50% (TCID_50_) in KC cells as previously described [38].

### Insect Species

Adults of both *Culicoides sonorensis* Wirth & Jones 1957 (PIRB -s-3 strain), and *Culicoides nubeculosus* Meigen 1830 (PIRB strain) from colonies held at The Pirbright Institute were used. Maintenance was as described previously [2], with the exception that the colonies were sustained by blood feeding through artificial membranes (Parafilm ™) over a heated reservoir (Hemotek, UK) filled with defibrinated horse blood from a commercial supplier (TCS Bioscience, Botolph Claydon, UK).

### Infection of *Culicoides* with BTV

Both *Culicoides* species were exposed to BTV by two routes, oral infection via a virus-infected blood meal or intrathoracic inoculation (ITI). For oral infection, approximately 500 adult *C. sonorensis* at 3 days post eclosion were exposed to a 3:1 horse blood:BTV tissue culture mixture at a calculated titre of 7.4 log_10_ TCID_50_/ml using a reservoir and heating unit (Hemotek, UK) at 37 °C and a Parafilm™ membrane. After 30 min exposure to the blood meal, individuals were immobilised under light anaesthesia with CO_2_. 150 fully engorged, blood-fed females were transferred into a cardboard pillbox (Watkins and Doncaster, Leominster, UK) and incubated in the dark at 25 °C, 80% relative humidity (RH) and fed *ad libitum* with 10% sucrose on a cotton pad refreshed daily for a period of 8 or 15 days, depending on the experiment. Additionally, 3-day-post eclosion adult female *Culicoides* were intrathoracically (IT) inoculated with BTV-4. 50 *Culicoides* were inoculated with ≤ 0.2 µl 6.4 log_10_ TCID_50_/ml BTV-4 MOR2009/07 using a pulled glass needle and Nanoject II microinjector (Drummond Scientific, NJ, USA) under light CO_2_ anesthesia. Inoculation site was either under the dorsal mesonotum or laterally between thoracic plates above the legs depending on individual presentation. Inoculated individuals were transferred to a cardboard pill box and incubated as for membrane-fed *Culicoides* for 5 or 8 days. For both oral and ITI treatments, post incubation surviving insects were killed by immersion into DPBS (Gibco™, Life Technologies, Inchinnan, UK) + 0.05% Tween® 20 (Sigma-Aldrich®, Gillingham, UK) for 20 min immediately prior to dissection. Mock-infected individuals were treated as above with oral-fed controls receiving a blood meal with no virus via Hemotek and ITI controls inoculated with the same volume of Schneider’s Drosophila media (Gibco™, Life Technologies, Inchinnan, UK) without virus.

### Dissection

Dissection was carried out under a stereomicroscope (Leica MZ60) using sterile 25G hypodermic needles for each individual (Fisher Scientific, Loughborough, UK). Individual *Culicoides* were placed in single drop of DPBS (Gibco™, Life Technologies, Inchinnan, UK) + 0.05% Tween® 20 (Sigma-Aldrich®, Gillingham, UK). The head was carefully removed, keeping the salivary glands attached, and transferred using the needle to a well in a 96-well flat-bottom microplate with 200 µl DPBS + 0.05% Tween 20. The DPBS was aspirated from the well using a pipette and discarded. 200 µl of 4% paraformaldehyde (PFA) in PBS (Thermo Scientific Chemicals, Inchinnan, UK) was added to each sample and the sample incubated for one hour at room temperature. After fixation, the 4% PFA was removed by aspiration and the samples rinsed with DPBS three times. Samples were stored in DPBS at + 4 °C until immunolabelling. The remaining body of each individual was placed in a sample 96-microtube plate (Qiagen, Manchester, UK) containing 200 µl RPMI (Gibco™, Life Technologies, Inchinnan, UK) + 2% penicillin / streptomycin (Gibco™, Life Technologies, Inchinnan, UK) + 2% amphotericin B (Sigma-Aldrich®, Gillingham, UK) and a 3 mm stainless steel bead (Dejay Distribution Limited Ltd, Launcestron, UK) for homogenisation. Plates were sealed with caps and bodies were homogenised in a Tissue Lyser (Qiagen, Manchester, UK) [41]. Homogenates were then topped up with RPMI + 2% penicillin/ streptomycin + amphotericin B to 1 mL and stored at + 4 °C until RNA extraction.

### Immunofluorescence Labelling and Imaging

Dissected and fixed salivary apparatus-head combinations were immunolabelled in 96-well flat-bottom microtiter plates for cellular tubulin, BTV structural proteins (VSPs) and/or the BTV non-structural protein 2 (NS2). Briefly, using a single channel 200 µl pipette, DPBS (Gibco™, Life Technologies, Inchinnan, UK) was removed, and tissue permeabilization was carried out by adding 200 µl of 0.5% Triton X-100 (Sigma-Aldrich®, Gillingham, UK) / DPBS magnesium (Mg) and calcium (Ca) free for 15 min. Triton was then removed as above followed by three washes with 200 µl of DPBS (Mg and Ca free) and a one-hour blocking step with 200 µl of blocking buffer (1% bovine serum albumin (Sigma-Aldrich®, Gillingham, UK) / 0.2% normal goat serum (Sigma-Aldrich®, Gillingham, UK) / DPBS Mg and Ca free. Next, blocking buffer was removed and 200 µl of a cocktail of specific primary antibodies (Table 2) diluted in blocking buffer was added. After 90 min of incubation at room temperature, primary antibodies were removed, organs washed three more times with DPBS as above, and 200 µl of appropriate secondary antibody cocktail (Table 2) diluted in blocking buffer was added and incubated at room temperature in the dark for 90 min. Next, organs were washed three times with DPBS. Where cell nuclei were to be stained, 40, 6-Diamidino-2-phenylindole (DAPI) (Life Technologies Limited, Paisley, UK) was added at this point and incubated for 30 min at the manufacturer’s recommended dilution, followed by three washes with ultra-pure water.

**Table 2:** List of antibodies used in this study for immunolabelling

**Antibody**	***Species* raised in**	**Target**	**Working Dilution**^**a**^	**Obtained from**
Orab1	Rabbit (polyclonal)	NS2 of BTV-1	1:2000	TPI^b^
Orab279	Guinea pig (polyclonal)	BTV-1 structural proteins	1:2000	TPI^b^
Anti-tubulin	Mouse (monoclonal)	α-tubulin	1:1000	Sigma-Aldrich® (T6199)
AlexaFluor™ 405	Goat	Mouse IgG (H + L)	1:250	Invitrogen™ (A48255)
AlexaFluor™ 488	Goat	Guinea pig IgG (H + L)	1:250	Invitrogen™ (A-11073)
AlexaFluor™ 568	Goat	Rabbit IgG (H + L)	1:250	Invitrogen™ (A-11036)
AlexaFluor™ 568	Goat	Mouse IgG (H + L)	1:250	Invitrogen™ (A-11031)

^a^diluted in blocking buffer

^b^from the Pirbright Orbivirus antibody collection

**Fig. 11:**
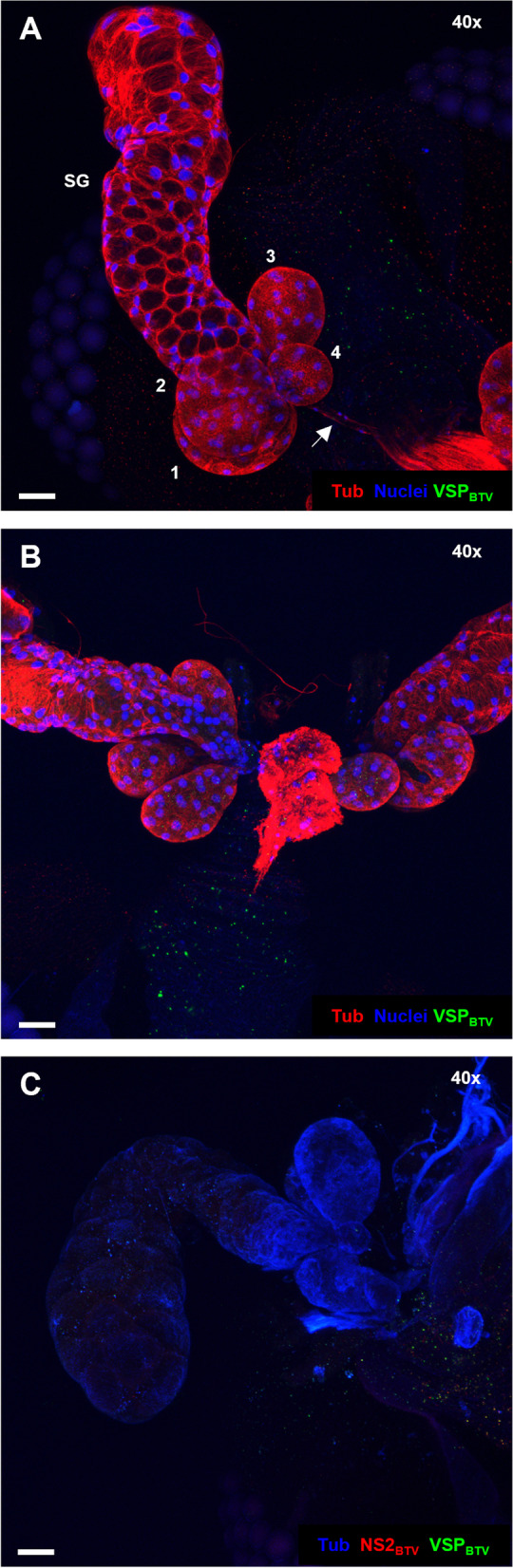
Representative images of immunolabelling background controls. **A**. Salivary apparatus of a *C. sonorensis* orally fed on BTV-spiked blood, but BTV negative. **B**. Salivary apparatus of a *C. sonorensis* fed on mock blood and, **C**. Salivary apparatus of a *C. sonorensis* mock infected by intrathoracic inoculation. In **A** and **B** cell nuclei are shown in blue (DAPI staining), and cellular tubulin is visualised in red (labelling with mouse anti-tubulin, and anti-mouse IgG AlexaFluor™ 568). Absence of green denotes absence of BTV structural proteins (VSPs) which were labelled with in-house antibody Orab279 and anti-guinea pig IgG AlexaFluor™ 488. In panel **C**, cellular tubulin is shown in blue (labelling with mouse anti-tubulin, and anti-mouse IgG AlexaFluor™ 405). Absence of red or green denotes absence of BTV non-structural protein 2 (NS2) or BTV VSPs, respectively. Viral protein NS2 was labelled with in-house rabbit antibody Orab1 and anti-rabbit IgG AlexaFluor™ 568; and viral VSPs were labelled with Orab279 followed by anti-guinea pig IgG AlexaFluor™ 488 as in panels **A** and **B**. In **A**, one main salivary gland (SG) with its respective four accessory glands (1 to 4) and the salivary duct (arrow) can be observed. All panels are maximum projections of at least 50 stacked plane images. Microscope magnification is shown on the top right corner and the scale bar represents 20 µm

To prevent destruction of organ structures, a gene-frame (25 µl, 10 mm x 10 mm; ThermoFisher Scientific, Loughborough, UK) was placed on glass microscope slides and filled with 30 to 40 µl of Vectashield® Hardset Mounting Medium (Vector Laboratories, Burlingame, CA, USA). Fine forceps were used to place the samples on the microscope slide. Using a stereomicroscope (Leica MZ60), positioning of the sample was assessed and corrected if needed. Finally, a square glass coverslip was glued to the gene-frame and organs were either visualized immediately or stored at + 4 °C for a maximum of three days before being imaged. Organs of mock-infected and dissected *C. sonorensis* were included as immunofluorescence background controls in all experiments (Fig. 11). Samples were imaged using a Leica SP8 CLSM confocal microscope (Leica Microsystems, Wetzlar, Germany).

### RNA Extraction and Viral Genome Detection and Quantification

Total RNA was extracted from 100 µl of each insect homogenate using the KingFisher Flex robotic extraction system (ThermoFisher Scientific, Loughborough, UK) using the MagMAX™ CORE Nucleic Acid purification kit (ThermoFisher Scientific, Loughborough, UK) kit as per manufacturer’s instructions. Six microlitres of each extracted RNA was tested by qRT-PCR targeting Segment 10 (Seg-10) of BTV as described by [15], but adapted for the SuperScript III platinum one-step qRT-PCR (Invitrogen™, Life Technologies, Inchinnan, UK). Viral genome copies were quantified using a 10-fold dilution series of BTV-1 Seg-10 RNA transcript as standard [33]. qRT-PCR results were plotted using GraphPad Prism software version 9.3.1 (GraphPad Software, San Diego, USA).

## Data Availability

Certain material used in this study can be made available from the senior author on reasonable request.
